# Clinical Evaluation of Javanica Oil Emulsion Injection Combined with the Radiotherapy in the Treatment of Esophageal Cancer: A Systematic Review and Meta-Analysis

**DOI:** 10.1089/acm.2018.0096

**Published:** 2019-05-09

**Authors:** Jiarui Wu, Mengwei Ni, Jialian Zhu, Kaihuan Wang, Dan Zhang, Shuyu Liu

**Affiliations:** Department of Clinical Chinese Pharmacy, School of Chinese Materia Medica, Beijing University of Chinese Medicine, Beijing, China.

**Keywords:** Javanica oil emulsion injection, radiotherapy, esophageal cancer, randomized controlled trials, meta-analysis

## Abstract

***Objectives:*** This meta-analysis aimed to assess the clinical effectiveness and safety of Javanica oil emulsion injection (JOEI) combined with the radiotherapy (RT) for treating esophageal cancer (EC).

***Design:*** A literature search was conducted for collecting the randomized controlled trials (RCTs) on EC treated by JOEI in the Cochrane Library, PubMed, Embase, the Chinese Biomedical Literature Database (SinoMed), the China National Knowledge Infrastructure Database, the China Science and Technology Journal Database (VIP), and the Wanfang Database from inception to February 4, 2017. The quality of the RCTs was evaluated by the Cochrane risk of bias assessment tool, and objective remission rate, performance status, adverse drug reactions (ADRs), 1-year survival rate, and 2-year survival rate were analyzed by Review Manager 5.3 and Stata 13.0 software.

***Results:*** A total of 11 RCTs with 909 participants were involved in this meta-analysis. The results showed that in comparison with RT alone, the JOEI combined with RT was associated with the better effects on improving objective remission rate (relative risk [RR] = 1.33, 95% confidence interval [CI 1.17–1.52], *Z* = 4.44, *p* < 0.00001), performance status (RR = 1.52, 95% CI [1.25–1.85], *Z* = 4.24, *p* < 0.00001), 1-year survival rate (RR = 1.37, 95% CI [1.17–1.60], *Z* = 3.86, *p* < 0.0001), and 2-year survival rate (RR = 1.36, 95% CI [1.09–1.70], *Z* = 2.68, *p* = 0.007). The differences between the two groups in objective remission rate, performance status, 1-year survival rate, and 2-year survival rate were statistically significant. Besides, the JOEI combined with RT could reduce the incidence of ADRs. Specifically, the statistically significant difference was detected between these two groups about leukopenia (RR = 0.39, 95% CI [0.25–0.61], *Z* = 4.19, *p* < 0.0001), radiation esophagitis (RR = 0.68, 95% CI [0.50–0.93], *Z* = 2.42, *p* = 0.02), thrombocytopenia (RR = 0.92, 95% CI [0.12–0.66], *Z* = 2.95, *p* = 0.003), and hemoglobin reduction (RR = 0.53, 95% CI [0.35–0.79], *Z* = 3.14, *p* = 0.002); however, there was no statistically significant difference for the outcome of nausea and vomiting (RR = 0.61, 95% CI [0.36–1.03], *Z* = 1.85, *p* = 0.06) between two groups.

***Conclusion:*** This meta-analysis indicated that the combination of JOEI and RT was associated with the more beneficial treatment for patients with EC compared with only receiving RT. However, more well-designed and multicenter RCTs should be carried out to confirm this finding because of the limitations of enrolled 11 RCTs.

## Introduction

Esophageal cancer (EC) is one of the common alimentary system cancers worldwide.^1,2^ According to the relative report, its mortality may increase to 728,945 by 2035 and its incidence in men is 3–4 times higher than in females.^3,4^ Due to dysphagia, the patients with EC usually suffer from hiccups, weight loss, and so on in early stages.^5,6^ Although the pathogeny of EC is still unclear, several chemical elements such as nitrosamines, the lack of nutrition, and some biologic factors are deemed to dangers.^7,8^ Currently, the treatment methods for EC were mainly radiotherapy (RT), surgery, chemotherapy, and so on.^9^ Among them, RT is a critical method in the treatment of EC, but adverse drug reactions (ADRs) would occur in treating, for instance, leukopenia, radiation esophagitis, nausea, and vomiting.^10,11^ Because of the patients' intolerance, these ADRs may cause the interruption of treatments. In recent years, the integrated treatment of EC has been gradually recognized, and Traditional Chinese Medicine (TCM) could achieve the therapeutic effects of improving clinical benefits and reducing ADRs of RT.^12,13^ EC belongs to dysphagia occlusion in TCM theory, which is considered as the occurrence of obstruction of esophagus caused by phlegm, blood stasis, and calculi.^14,15^

As one of the most active TCM preparations against cancers, Javanica oil emulsion injection (JOEI) has been authorized by the China Food And Drug Administration to cure a wide range of solid tumors, including EC, gastrointestinal cancer, lung cancer, and ovarian cancer.^16–20^ It is extracted from *Brucea* Jen emulsion petroleum ether and purified soybean lecithin.^21,22^ Some previous researches indicated that the oleic acids and linoleic acids, two major active ingredients of JOEI, related with the anticancer effect by inhibiting and killing the cancer cells in the G0, G1, S, G2, and M phases and DNA topoisomerases I (TOP1) or II (TOP2).^23–25^ Presently, there were two meta-analyses concerning about JOEI combined with RT for treating EC.^26,27^ Neither of them evaluate the quality of the articles and the stability of the results. Therefore, the authors performed the present meta-analysis to assess the effectiveness and safety of JOEI for EC objectively.

## Materials and Methods

### Literature search

The Cochrane Library, PubMed, Embase, SinoMed, China National Knowledge Infrastructure, VIP, and the Wanfang Database were systematically searched to identify randomized controlled trials (RCTs) concerning JOEI for EC from inception to February 4, 2017. The authors adopted the search strategies for each database with the combination of mesh terms and free-text terms. The manual search and gray literature search were carried out to identify the potential RCTs, including related meta-analysis, references of included RCTs, Masters' and Doctors' articles, and some ongoing research study websites.

Take the strategy of PubMed as example:

#1 “Esophageal Neoplasms”[Mesh]
#2 “Esophageal Neoplasms*”[Title/Abstract] OR “Esophagus Neoplasm*”[Title/Abstract] OR “Esophagus Cancer*”[Title/Abstract] OR “Esophageal Cancer*”[Title/Abstract] OR “Cancer of Esophagus” [Title/Abstract]
#3 #1 OR #2
#4 “Javanica oil emulsion injection”[Title/Abstract] OR “Yadanzi injection”[Title/Abstract] OR “Yandanzi” [Title/Abstract]OR “Brucea javanica”[Title/Abstract]
#5 #3 AND #4

### Inclusion and exclusion criteria

RCTs were eligible if they were corresponded with the following criteria: (1) The type of articles was RCTs that focused on JOEI for EC without the restriction on the blinding method. (2) All patients were diagnosed as EC through gastrointestinal endoscopy, digestive tract endoscopy, chest computed tomography, or histopathologic diagnosis. In addition, the Karnofsky performance score (KPS) was above 60 in all patients to ensure that the long-term outcomes could be involved. The age, gender, course of disease, race, and severity of the disease were not limited. (3) Intervention for control group was RT, intensity modulated radiation therapy, three dimensional conformal radiotherapy, and conventional fractionated radiotherapy. Patients in experimental group were treated by RT plus JOEI. (4) The primary outcomes were objective remission rate and performance status. The criteria of objective remission rate abide conform to Solid Tumor Short-Term Effectiveness Criteria established by the World Health Organization.^28^ Objective remission rate = (complete remission number + partial remission number)/total number of patients × 100%. With regard to the improvement of performance status, it was calculated as KPS, if KPS increased 10 points were regarded as improvement of performance status. Secondary outcomes included the ADRs, survival rate, and immunologic function.

RCTs that met the following criteria were excluded: (1) Repeated published data, the full text was not available, pharmacology experiments, or individual cases. (2) TCM decoction, acupuncture, or other TCM treatments in RCTs. (3) Patients suffered from other diseases. (4) RCTs did not describe or meet the standard of efficacy. (5) RCTs were with high risk random method. (6) RCTs did not report the data of objective remission rate, performance status, and ADRs.

### Data extraction and quality assessment

Data extraction was independently performed by two reviewers (J.W. and M.N.) and by utilizing NoteExpress software (Wuhan University Library, Wuhan, China) to filter the duplication and the irrelevant literatures by reading titles and abstracts. The remaining articles were browsed full text to determine whether they met the inclusion criteria or not. The following information was collected in this meta-analysis: the first author's name, publication year, number of patients, gender, age, intervention, course of treatment, and the data of outcome. The Cochrane risk of bias assessment tool was adopted for quality assessment in this meta-analysis. And it would assess the quality of RCTs from six aspects, which contained selection bias, performance bias, detection bias, attrition bias, reporting bias, and other bias with “high,” “unclear,” and “low” three levels.^29^ It was essential to have a discussion with a third researcher (K.W.) when two reviewers emerged with different results.

Medical ethics was not obligation for this meta-analysis because the study was a systematic review of published RCTs.

### Statistical analysis

Review Manager 5.3 software (Cochrane Collaboration, Oxford, United Kingdom) and Stata 13.0 software (Stata Corporation, College Station, TX) were applied to analyze the data for this meta-analysis. The relative risk (RR) was calculated for the binary variable, the mean difference was adopted for continuous variable, and each outcome was presented with 95% confidence interval (CI). The heterogeneity among RCTs was counted by the chi-squared and *I*^2^ tests. If *p* > 0.1, *I*^2^ < 50%, the fixed effect model was recommended. Otherwise, the random effect model was used.^30^ Subgroup analysis should be performed in consideration of clinical or methodological heterogeneity. For example, different doses or courses of interventions. Sensitivity analysis was achieved by Stata13.0 software to evaluate the stability of the results.^31^ In addition, the Funnel plot, Begg's, and Egger's tests were administered to detect publication bias. In Begg's and Egger's tests, *p* < 0.05 indicated a certain publication bias among included RCTs.^32^

## Results

### Study characteristics

**FIG. 1.**
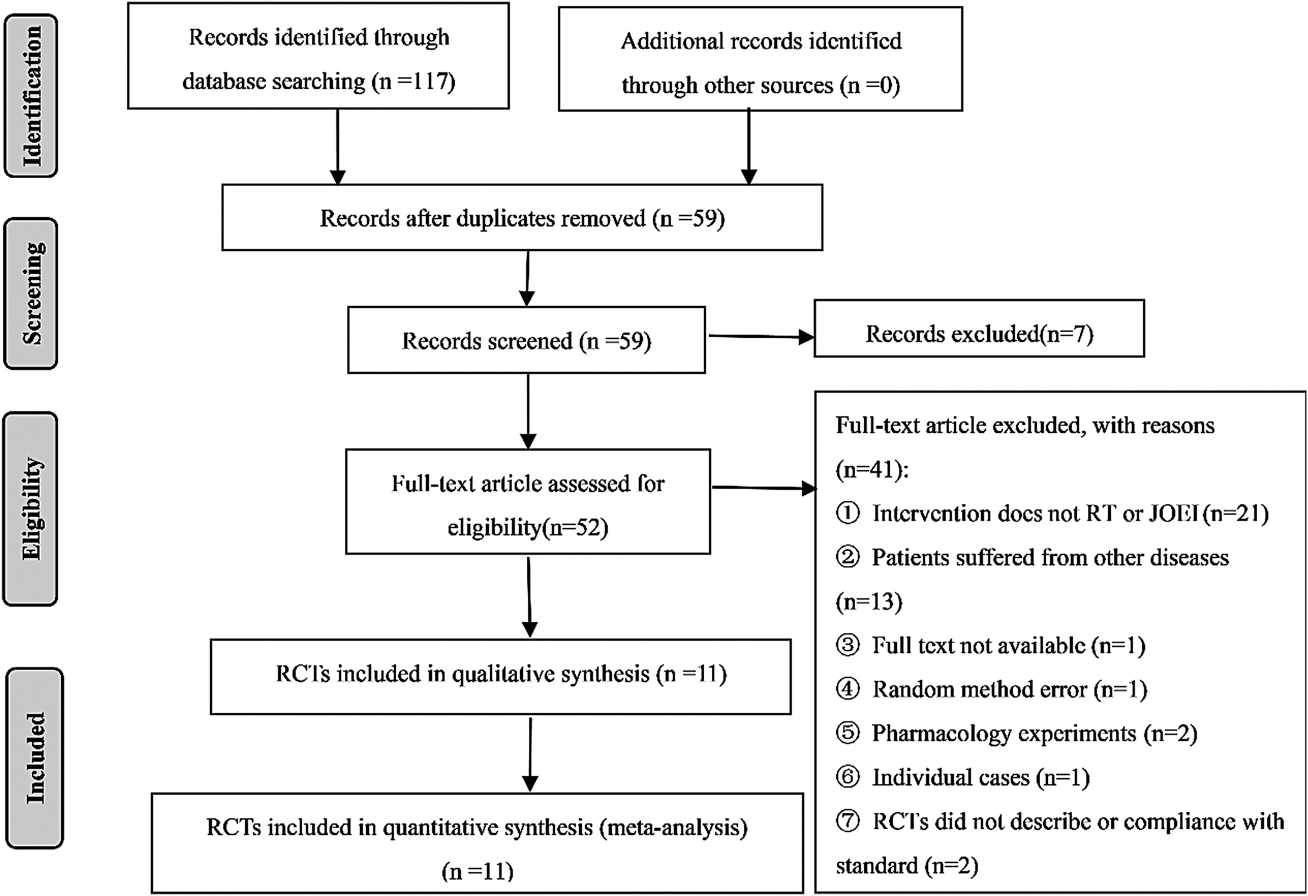
Flow chart of literature search. RCTs, randomized controlled trials.

Initially, 117 potentially relevant articles were retrieved, after screening titles and abstracts to exclude duplication and irrelevant articles; 52 articles remained for full-text review. Furthermore, 41 articles were ruled out for reasons described in Figure 1, and 11 RCTs were eligible in this meta-analysis ultimately. The 11 RCTs enrolled 909 patients, in which 463 cases were in the experimental group and 446 cases were in the control group. The age range of patients was from 30 to 81 years old. Among them, male patients accounted for 69.42%. The majority dosage of JOEI was 30 mL/day, and the course of treatment was more than 18 days (Table 1).

**Table 1.** The Basic Characteristics of the Included Randomized Controlled Trials

*Study ID*	*Sex (M/F)*	*Avg. age*	N *(E/C)*	*Therapy of experiment*	*Therapy of control*	*Course (days)*	*Outcomes*
Feng^34^	35/25	50–81	30/30	JOEI 30 mL + RT	RT	21	①②
Li et al.^35^	36/14	62	25/25	JOEI 30 mL + IMRT	IMRT	21	①②④
Jia et al.^36^	116/32	55.5	76/72	JOEI 30–50 mL + RT	RT	30	①②③④⑤
Kong et al.^37^	47/13	54.5	30/30	JOEI 30 mL + RT	RT	21	①②④
Liu and Zhu^38^	39/17	30–78	28/28	JOEI 30 mL + RT	RT	21	①②③
He et al.^39^	63/7	52–78	35/35	JOEI 30 mL + RT	RT	18–27	①②③
Qi and Zhang^40^	68/42	47–78	61/49	JOEI 20–30 mL + TDCRT	TDCRT	30	①②③
Sheng et al.^41^	62/48	/	55/55	JOEI 30 mL + RT	RT	28	①②③
Jiang and Huo^42^	41/28	39–68	35/34	JOEI 30 mL + RT	RT	42–49	①②
Li et al.^43^	37/19	29–74	28/28	JOEI 30 mL + TDCRT	TDCRT	21	①②④
Liu and Zuo^44^	87/33	40–83	60/60	JOEI 30 mL + CFRT	CFRT	21	①②④

①, objective remission rate; ②, ADR; ③, life status improvement rate; ④, survival rate; ⑤, immunologic function.

ADR, adverse drug reaction; C, control group; CFRT, conventional fractionated radiotherapy; E, experiment group; F, female; IMRT, intensity modulated radiation therapy; JOEI, Javanica oil emulsion injection; M, male; RT, radiotherapy; TDCRT, three dimensional conformal radiotherapy.

**FIG. 2.**
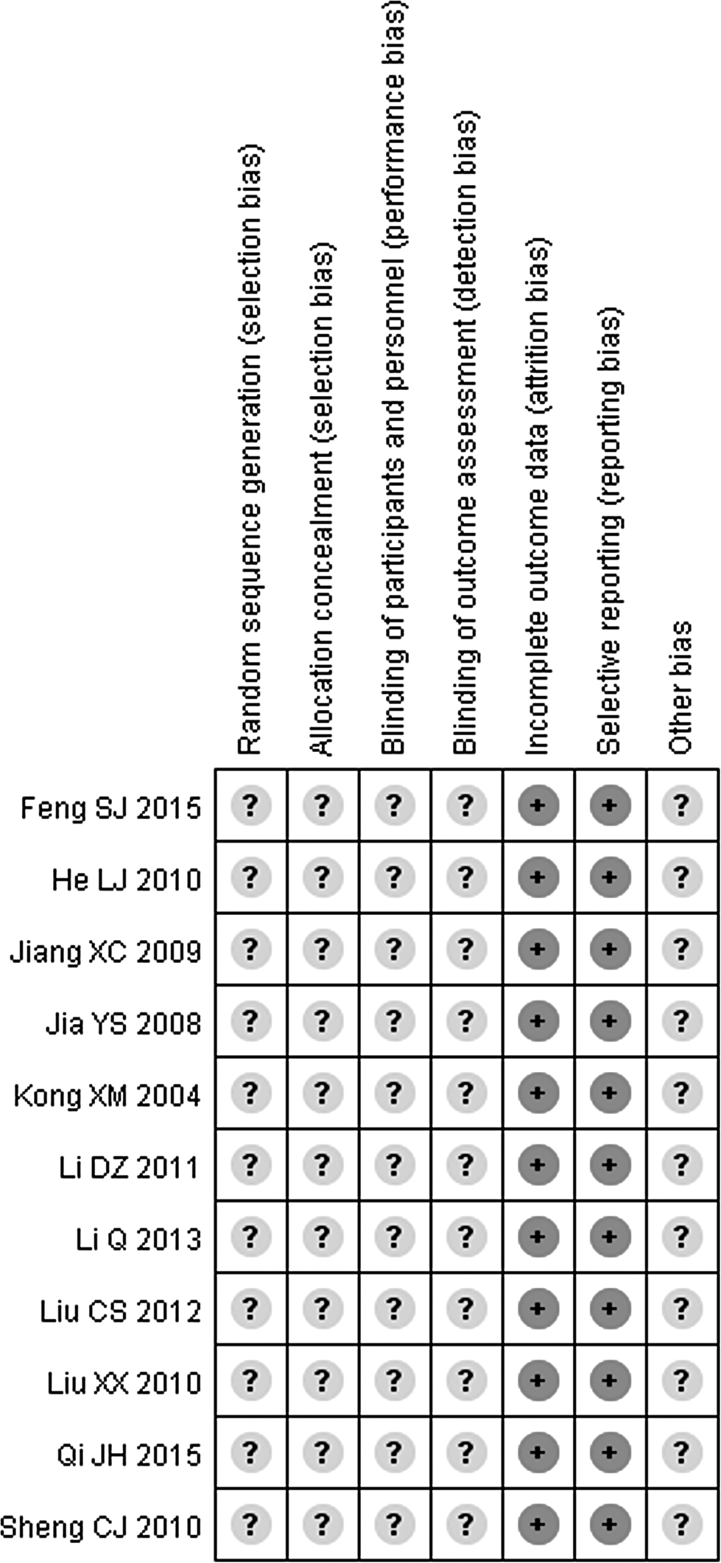
Risk of bias graph. Low risk is indicated with a *question mark,* and unclear bias is indicated with a *plus sign.*

As Figure 2 presented, the Cochrane risk of bias assessment tool was applied to evaluate the quality of the RCTs. None of them mentioned the specific grouping method, the concealment of random sequences, and the implementation of blinding, so the selective bias, the performance bias, and the detection bias were judged as “unclear.” Moreover, because of the inexistence of selective reporting and case detachment in all RCTs, their attrition bias and reporting bias were assessed as “low risk.” Owning to the RCTs did not describe the bias of other aspects; the others bias was remarked as “unclear.”

### Outcomes

#### Objective remission rate

**FIG. 3.**
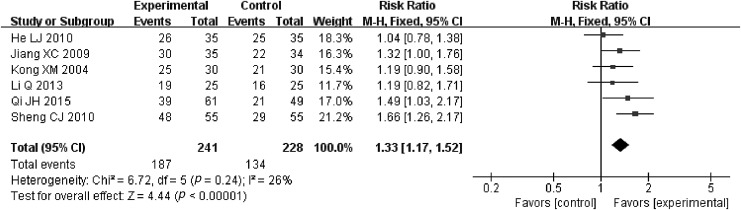
Meta-analysis in objective remission rate between JOEI + RT and RT. CI, confidence interval; JOEI, Javanica oil emulsion injection; RT, radiotherapy.

A total of six RCTs described the statistics of objective remission rate.^33,36,38–41^ Small heterogeneity was detected among them (*p* = 0.24 > 0.1, *I*^2^ = 26% <50%), so the fixed effect model was used. As displayed in Figure 3, the result of meta-analysis manifested that compared with RT, JOEI plus RT could increase the objective remission rate to about 23%. The difference between the two groups was statistically significant (RR = 1.33, 95% CI [1.17–1.52], *Z* = 4.44, *p* < 0.00001).

#### Performance status

**FIG. 4.**
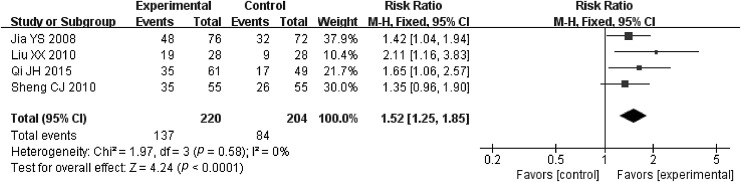
Meta-analysis in Performance Status Improvement Rate between JOEI + RT and RT. CI, confidence interval; JOEI, Javanica oil emulsion injection; RT, radiotherapy.

Four RCTs listed the details about performance status.^35,37,39,40^ Heterogeneity was moderate among the RCTs (*p* = 0.58 > 0.1, *I*^2^ = 0% <50%); hence a fixed effect model was chosen. The result of meta-analysis showed that compared with only undergoing RT treatment, JOEI associated with RT can enhance the performance status of patients with EC to about 59%. In addition, there was a statistically significant difference between the two groups (RR = 1.52, 95% CI [1.25–1.85], *Z* = 4.24, *p* < 0.00001) (Fig. 4).

#### Adverse drug reactions

All RCTs in this meta-analysis recorded the ADRs; their study mainly covered the leukopenia, radiation esophagitis, thrombocytopenia, hemoglobin reduction, nausea, and vomiting.

##### Leukopenia

**FIG. 5.**
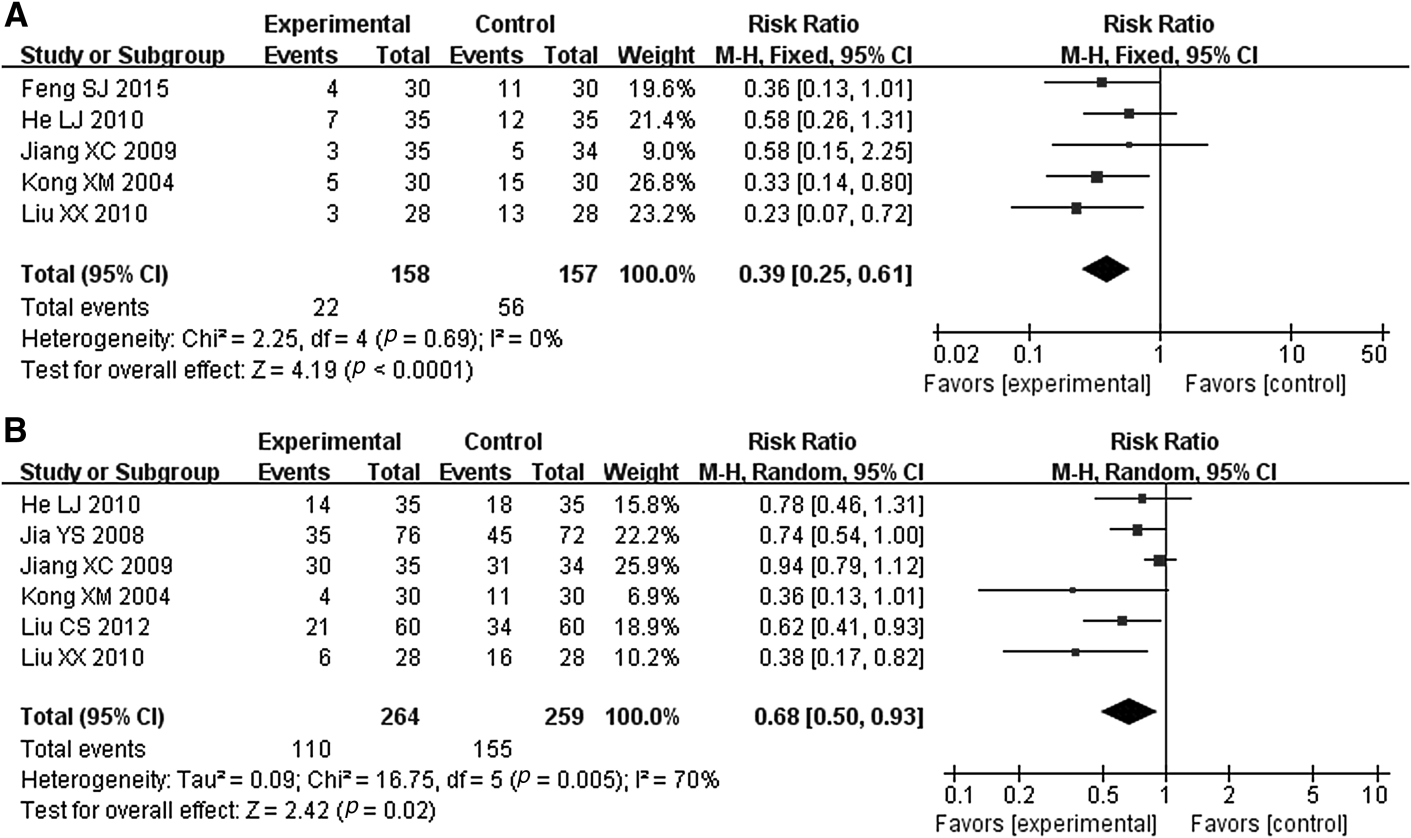
Meta-analysis in adverse drug reactions in between JOEI + RT and RT. **(A)** Leukopenia, **(B)** radiation esophagitis. CI, confidence interval; JOEI, Javanica oil emulsion injection; RT, radiotherapy.

Five RCTs recorded the leukopenia.^34,36–38,41^ The heterogeneity was small (*p* = 0.69 > 0.1, *I*^2^ = 0% <50%); thus, the fixed effect model was implemented. Meta-analysis revealed that compared with the control group only using RT, JOEI with RT can reduce leukopenia. Besides, the statistically significant difference was detected between these two groups (RR = 0.39, 95% CI [0.25–0.61], *Z* = 4.19, *p* < 0.0001) (Fig. 5A).

##### Radiation esophagitis

Six RCTs noted information about the radiation esophagitis.^35–38,41,43^ Heterogeneity was high among the RCTs (*p* = 0.0005 > 0.1, *I*^2^ = 70%> 50%); therefore, the random effect model was used. The result of meta-analysis indicated that compared with RT alone, JOEI combined with RT can reduce radiation esophagitis in EC patients, and the difference between the two groups was statistically significant (RR = 0.68, 95% CI [0.50–0.93], *Z* = 2.42, *p* = 0.02) (Fig. 5B).

As displayed in Table 2, other ADRs mainly included thrombocytopenia,^34,37^ hemoglobin reduction,^34,37^ nausea, and vomiting.^34,36,38^ Compared with RT alone, the combination of RT and JOEI had a better effect on relieving thrombocytopenia and hemoglobin reduction; the difference was statistically significant between two groups (*p* < 0.05). However, there was no statistically significant difference for the outcome of nausea and vomiting between two groups (*p* > 0.05).

**Table 2.** Meta-Analysis of Other Adverse Drug Reactions

*Outcomes*	*Number of RCTs*	*Model*	*RR [95% CI]*	Z	p
Thrombocytopenia	2	Fixed effect	0.92 [0.12–0.66]	2.95	0.003
Hemoglobin reduction	2	Fixed effect	0.53 [0.35–0.79]	3.14	0.002
Nausea and vomiting	3	Fixed effect	0.61 [0.36–1.03]	1.85	0.06

CI, confidence interval; RCT, randomized controlled trial; RR, relative risk.

#### Survival rate

##### One-year survival rate

**FIG. 6.**
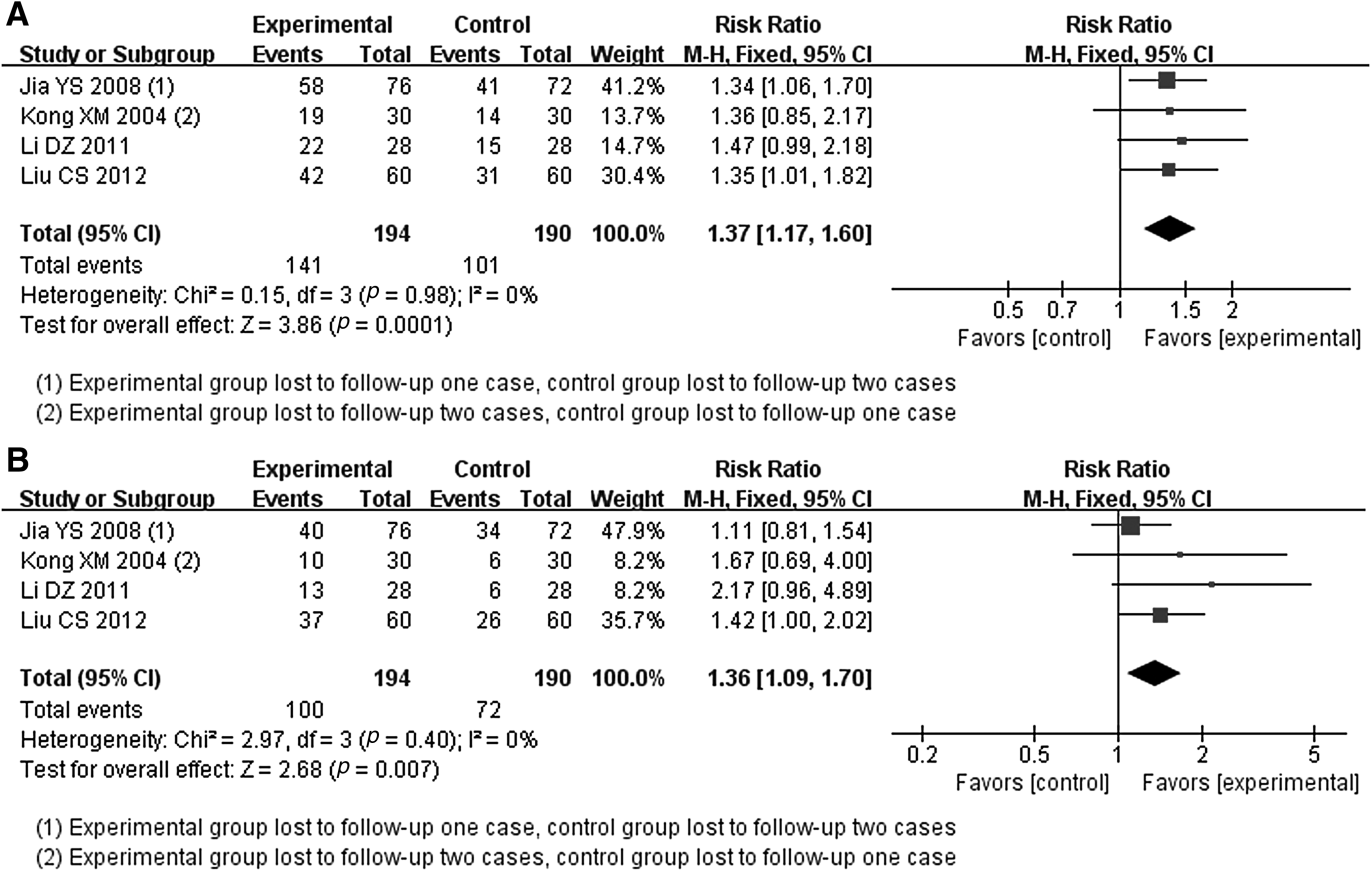
Meta-analysis in survival rate between JOEI + RT and RT. **(A)** One-year survival rate, **(B)** 2-year survival rate. CI, confidence interval; JOEI, Javanica oil emulsion injection; RT, radiotherapy.

One-year survival rate had appeared in four RCTs.^35,36,42,43^ The heterogeneity test (*p* = 0.98 > 0.1, *I*^2^ = 0% <50%) texted the existence of small heterogeneity; thus, the fixed effect model was applied. Meta-analysis signified that there was significant difference between the two groups in this outcome. The 1-year survival rate in JOEI combined with RT group was associated with a higher 1-year survival rate (RR = 1.37, 95% CI [1.17–1.60], *Z* = 3.86, *p* < 0.0001) (Fig. 6A).

##### Two-year survival rate

Four RCTs reported the 2-year survival rate.^35,36,42,43^ The heterogeneity test (*p* = 0.40 > 0.1, *I*^2^ = 0% <50%) measured the heterogeneity which was small, thus the fixed effect model was performed. Meta-analysis discovered that JOEI cooperated with RT was associated with a higher 2-year survival rate. Moreover, difference between groups was statistically significant (RR = 1.36, 95% CI [1.09–1.70], *Z* = 2.68, *p* = 0.007) (Fig. 6B).

### Immunologic function

There was only one RCT^35^ that recorded the T lymphocyte subsets comparing the two groups; thus, the meta-analysis performed a qualitative description for this outcome. The specific results are shown in Table 3.

**Table 3.** Meta-Analysis of Immunologic Function

*Outcomes*	*E*		*C*		p
	*Mean value*	*Standard deviation*	*Mean value*	*Standard deviation*	
CD3^+^/%	53.16	4.82	50.20	8.41	<0.05
CD4^+^/%	32.21	5.73	26.10	5.13	<0.05
CD8^+^/%	24.10	3.16	27.98	4.42	<0.05
CD4^+^/CD8^+^	1.38	0.34	1.06	0.29	<0.05

E, experiment group; C, control group.

### Sensitivity analysis

**FIG. 7.**
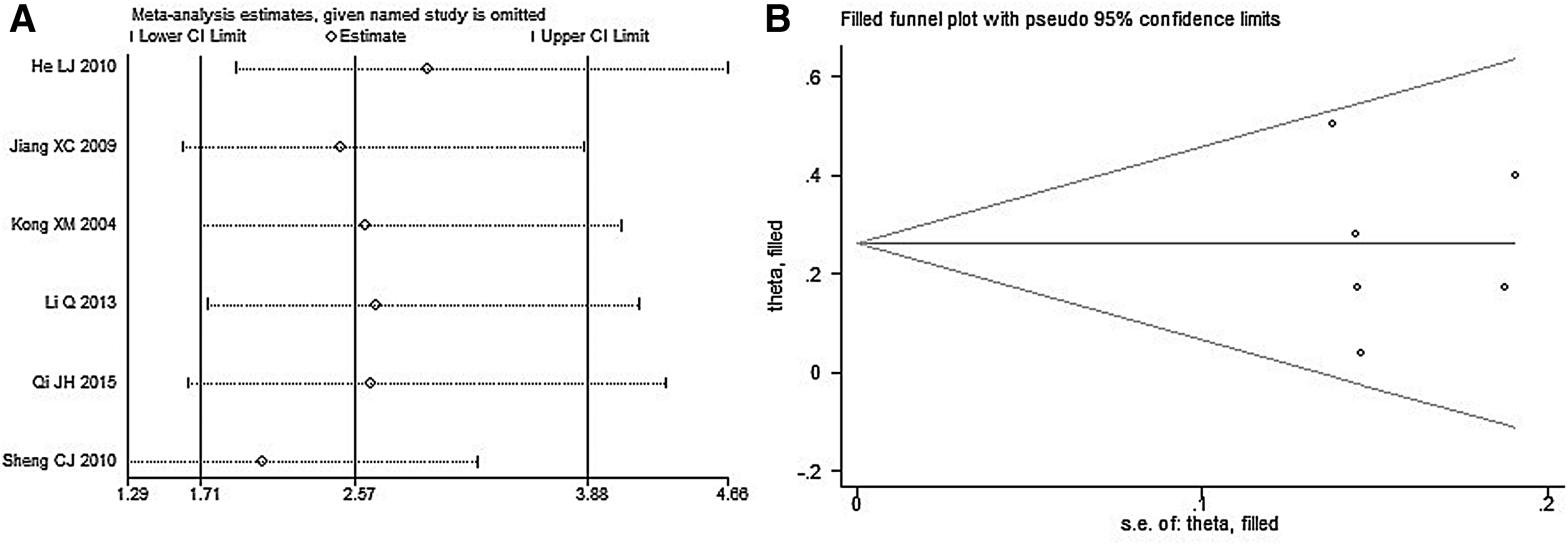
Sensitivity analysis and Funnel plot of objective remission rate. **(A)** Sensitivity analysis of objective remission rate, **(B)** Funnel plot of objective remission rate.

Sensitivity analysis was conducted for objective remission rate. As shown in Figure 7A, the results were not changed by combining effect, indicating that the results of this meta-analysis were steady.

### Evaluation of publication bias

The results of the Begg's test (*p* = 0.707 > 0.05) and Egger's test (*p* = 0.922 > 0.05) demonstrated that there were no obvious publication biases among included RCTs, and the distribution of RCTs was relatively symmetrical as well, as shown in Figure 7B.

## Discussion

In the present meta-analysis, the authors discussed the clinical effectiveness and safety of JOEI for treating EC. The results illustrated that the combination of JOEI based on RT had a better therapeutic effect for patients with EC. It can be reflected in the following aspects: JOEI combined with RT can not only ameliorate objective remission rate, performance status, 1-year survival rate, and 2-year survival rate but also relieve the ADRs caused by RT such as leukopenia, radiation esophagitis, thrombocytopenia, and hemoglobin reduction.

As ranking sixth among cancer worldwide, EC can appear in any part of the tube, and more than 450,000 cases are diagnosed with EC per year.^44,45^ Because of the atypical symptoms at earlier stages, it is always diagnosed at later stages.^46–48^ Besides, dysphagia is the most common symptom for patients with advanced EC.^49^ Owing to its complication, poor general conditions, and advanced age, the therapy of surgery or chemotherapy is often limited. Moreover, the 5-year overall survival rate for patients with EC is only 0%–20%, which is still poor after RT treatment alone.^50–53^ RT combined with TCM can not only improve the sensitivity of tumor cells to radiation but also reduce the ADRs of RT.^54^

Bencao Gangmu Shiyi recorded that *Brucea* Jen emulsion medicine in taste is bitter and in properties is cold. It has the effect of clearing heat to dry, killing parasites, and removing toxic substances.^55–57^ The mechanism of the JOEI may inhibit the activity of topoisomerase, blocking the proliferation of cancer cells, killing and impeding the cancer cells.^58^ JOEI could lead cancer cell death by both caspase-3 and caspase-9 or inhibition of NF-kappa B and Cyclooxygenase-2.^59^ JOEI is remarkable of its antitumor activity, and other pharmacologic experiment noted that JOEI can inhibit the proliferation of cancer cells by inhibiting the DNA synthesis.^60–63^

There were three prior meta-analyses^26,27,64^ on the treatment of EC with the application of JOEI, which contained two traditional meta-analyses.^26,27^ Among them, one was published in 2015, and two were published in 2016. In comparison, their meta-analysis has following advantages: First, this meta-analysis renewed the literature retrieval time and applied the search strategy of combining keywords and free-text words for a more comprehensive retrieval; for instance, two of RCTs included in this meta-analysis were published after 2015, while the lasted times for RCTs in the two prior meta-analyses were in 2013. Second, in this meta-analysis, all RCTs strictly conformed to inclusion and exclusion criteria to ensure the consistency of the baseline and reduce the clinical heterogeneity. Third, Stata 13.0 and RevMan 5.3 software were applied to analyze data, as well as examine the stability of the results through sensitivity analysis. Finally, outcomes of this meta-analysis not only concerned the objective remission rate and performance status but also the ADRs, which can effectively respond to the effectiveness of treatment for the patients with EC.

### Limitations

This meta-analysis also has a few limitations. First, the quality of the 11 RCTs enrolled in their study was not high. RCTs were all in Chinese and did not refer to the specific random grouping method; meanwhile the blind method and the covert grouping were not applied as well. Second, RCTs were mainly distributed at the bottom of the funnel, indicating a lack of a large sample of RCTs, which may increase the impact of treatment. Third, the survival rate is an important indicator for tumor treatment; however, because of the small number of included RCTs that mentioned survival rate the authors regarded it as a secondary outcome and RCTs also lacking of long-term outcomes, such as 3-year survival rate, follow-up data, and so on.

To provide the high-quality evidence, their study suggested that RCTs should pay more attention to long-term outcomes like survival rate. Furthermore, the quality of the RCTs should be improved, and clinician ought to emphasize the random methods, concealment of random sequence, implementation of blinding, and so on.

## Conclusions

In summary, the results of this meta-analysis illustrated that JOEI combined with RT had a better clinical effectiveness for EC. Nevertheless, the results of this meta-analysis need to be further validated by multicenter and larger sample RCTs (Supplementary Table S1).

## Supplementary Material

Supplemental data
